# Combination of the c-Met Inhibitor Tivantinib and Zoledronic Acid Prevents Tumor Bone Engraftment and Inhibits Progression of Established Bone Metastases in a Breast Xenograft Model

**DOI:** 10.1371/journal.pone.0079101

**Published:** 2013-11-18

**Authors:** Sara Previdi, Federica Scolari, Rosaria Chilà, Francesca Ricci, Giovanni Abbadessa, Massimo Broggini

**Affiliations:** 1 Laboratory of Molecular Pharmacology, Department of Oncology, IRCCS - Istituto di Ricerche Farmacologiche “Mario Negri”, Milan, Italy; 2 ArQule, Inc., Woburn, Massachusetts, United States of America; Stanford University, United States of America

## Abstract

Bone is the most common metastatic site for breast cancer. There is a significant need to understand the molecular mechanisms controlling the engraftment and growth of tumor cells in bone and to discover novel effective therapeutic strategies. The aim of this study was to assess the effects of tivantinib and Zoledronic Acid (ZA) in combination in a breast xenograft model of bone metastases. Cancer cells were intracardially implanted into immunodeficient mice and the effects of drugs alone or in combination on bone metastasis were evaluated by *in vivo* non-invasive optical and micro-CT imaging technologies. Drugs were administered either before (preventive regimen) or after (therapeutic regimen) bone metastases were detectable. In the preventive regimen, the combination of tivantinib plus ZA was much more effective than single agents in delaying bone metastatic tumor growth. When administered in the therapeutic schedule, the combination delayed metastatic progression and was effective in improving survival. These effects were not ascribed to a direct cytotoxic effect of the combined therapy on breast cancer cells *in vitro*. The results of this study provide the rationale for the design of new combinatorial strategies with tivantinib and ZA for the treatment of breast cancer bone metastases.

## Introduction

Bone is the most common metastatic site in breast cancer patients [1]. Bone metastases can be associated with pain, pathological fractures, nerve compression syndromes, hypercalcemia and can dramatically decrease the patient’s quality of life and shorten survival. [2], [3], [4], [5]. Skeletal complications can be managed locally by surgery or radiotherapy or systemically with hormonal agents, cytotoxics and analgesics, but these treatments don’t change patient prognosis. Therefore the development of bone metastasis represents one of the main problems in the management and treatment of patients with breast cancer. A better understanding of the mechanisms at the basis of breast cancer cells dissemination and colonization to the bones would foster the identification of key molecular pathways regulating the metastatic process and might provide new insights for the development of targeted treatments.

The formation of bone metastases is a multistep event regulated by a multitude of cellular and molecular interactions between cancer cells and host microenvironments. Combined approaches aimed at targeting both the “seeds” (tumor cells) and the “soil” (bone microenvironment) could lead to potentially succesfull therapeutic strategies for the treatment of advanced breast cancer.

Among different intracellular signalling transduction pathways, the hepatocyte growth factor (HGF) HGF/MET system is a key driver of oncogenesis and tumor progression in several human malignancies [6], [7], [8]. We have previously shown the involvement of the HGF/MET system in tumor-bone interaction contributing to human breast cancer metastasis [9]. We have also reported the effectiveness of MET inhibition, by both a MET inhibitor, tivantinib, and a specific short hairpin RNA (shRNA) against MET, in a mouse model of human breast cancer, in delaying the onset and progression of bone metastases. Tivantinib, in particular exhibited a dose-dependent antimetastatic activity in vivo, and at the 120 mg/kg dose, which was suboptimal in reducing subcutaneous tumor growth, induced significant inhibition of metastatic growth of breast cancer cells in bone and a clear reduction of tumor-induced osteolysis. [10]. Altogether our previously published data strongly suggest that targeting MET,might have a therapeutic value in the treatment of bone metastases from breast cancer.

Tivantinib (ARQ 197) is a selective, oral, non-ATP-competitive, small-molecule inhibitor of the MET receptor tyrosine kinase. Tivantinib demonstrated antiproliferative activity in several human cancer cell lines that constitutively express MET (IC_50_ = 0.30 to 0.66 µM), and showed significant antitumor activity in multiple xenograft tumor mouse models [11]. Moreover, the *in vivo* preclinical antimetastatic activity of tivantinib has been assessed in an orthotopic murine model of human colon carcinoma and in a humanized mouse model of breast cancer bone metastasis [2], [12], [13], [14].

Tivantinib is currently in clinical trials as a single agent and in combination with standard chemotherapies in different solid tumors [15], [16], [17], [18], [19], [20]. Overall, the most recent available data have shown promising results suggesting that tivantinib might be well tolerated and might have activity either alone or in combination with anticancer agents acting against other targets in patients with different tumors. Of particular interest are the results of a randomized placebo-controlled phase II study in patients with unresectable hepatocellular carcinoma pretreated with systemic therapy, where tivantinib induced a longer median time to progression in patients with high MET expression [21].

In addition to the tumor cells, also the osteoclasts in the host microenvironment play a pivotal role in the pathogenesis and sequelae of bone metastases. Osteoclasts cause bone resorption, which provides the spaces in which cancer cells grow as well as the release of various growth factors from bone matrix essential for tumor growth [22]. Bisphosphonates are potent inhibitors of osteoclast-mediated bone resorption and reduce significantly the frequency of skeletal-related events [23], [24], [25], [26]. Additionally, there is an exciting body of evidence suggesting that those drugs may have direct anti-tumor effects that may be exploited to prevent or delay the development of bone metastases [27], [28]. Their ability to induce apoptosis, inhibit tumor cell adhesion, invasion, and proliferation of human tumor cell lines has been demonstrated in numerous *in vitro* and *in vivo* studies [29], [30]. Moreover, there are pre-clinical data showing that the combination of bisphosphonates with chemotherapeutic agents can significantly increase the anti-tumor effects compared to single agents. [31]. In the present study, we evaluated the potential preventive and therapeutic efficacy of a dual strategy aimed at inhibiting the “vicious cycle” of bone metastases both on the tumor and the bone. To this aim, we investigated the effects of tivantinib against tumor-cells, in combination with ZA, against the bone metastatic environment, in an experimental model of bone metastases from breast cancer.

## Materials and Methods

### Cell Lines and Culture Conditions

The bone-seeking clone 1833/TGL, derived from the parental MDA-MB231 human breast cancer cell line was kindly provided by Dr. J. Massaguè (Memorial Sloan-Kettering Cancer Center, New York, NY, USA) [32]. The cells were cultured in DMEM (Dulbecco Modified Eagle’s Medium, Biowest), supplemented with 10% fetal bovine serum (Fetal Bovine Serum, Clontech) and 1% L-glutamine (Biowest), in a humidified CO_2_ incubator at 37°C.

### Reagents

Tivantinib [(-)-trans-3-(5,6-dihydro-4*H*-pyrrolo[3,2,1-ij]quinolin-1-yl)-4(1*H*-indol-3-yl)pyrrolidine-2,5-dione] was synthesized by ArQule, Inc. (Woburn,MA).

The commercially available clinical preparation of ZA [1-hydroxy-2-(1H-imidazole-1-yl)ethylidene-bisphosphonic acid], Zometa® (Novartis Pharma AG Basel, Switzerland) was used for these studies.

### In vitro Cytotoxic Assay

Exponentially growing 1833/TGL cells were seeded at 3×10^3^ cells/well in 96-well plates (Corning Glass Works, New York, NY, USA) and, after 24 hours, were treated with increasing concentrations of tivantinib and ZA alone or in combination. The microplates were incubated at 37°C and, at the end of the treatment period, MTS reagent (Promega, Madison, WI) was added to each well and the plates were incubated at 37°C for 4 hours in 5% CO_2_. Cell proliferation was evaluated by measuring the absorbance at 490 nm using infinite M200 microplate reader (TECAN, Switzerland). IC_50_ values were calculated by using non-linear regression method.

### In vitro Wound-healing Assay

1833/TGL cells were plated at the concentration of 7.5×10^4^ cells/mL in 12-wells plates (Becton Dickinson Labware). Upon confluence, a single wound was created in the center of the cell monolayers by gentle removal of the attached cells with a sterile plastic pipette tip. Cellular debris were removed by washing with PBS and then complete medium with or without tivantinib (0.4 µM) and/or ZA (1 µM) was added to each wells. The data are expressed as the percentage of wound closure and plotted against the hours after wounding. The experiments were performed in duplicate, each consisting of three replicates. Details are reported in Methods S1.

### Animals and Ethics Statement

Female athymic nude (nu/nu) 4–6 week-old mice (Harlan-Italy Udine, Italy) were used for these experiments. Mice were maintained in a temperature-controlled room with a 12-hour alternating light-dark cycle under specific pathogen-free conditions with food and water provided *ad libitum.* Procedures involving animals and their care were conducted in conformity with institutional guidelines that are in compliance with national (Legislative Decree 116 of January 27, 1992, Authorization n.169/94-A issued December 19, 1994, by Ministry of Health) and international laws and policies (EEC Council Directive 86/609, OJ L 358. 1, December 12, 1987; Standards for the Care and Use of Laboratory Animals, United States National Research Council, Statement of Compliance A5023-01, November 6, 1998).

The study protocol was approved by the IRCCS – Istituto di Ricerche Farmacologiche Mario Negri, internal Ethical Committee. The general health status of the animals was monitored daily. Tumor cell implants were performed under 4% isofluorane anesthesia, and all efforts were made to minimize suffering.

### Experimental Subcutaneous Xenograft Model

*In vivo* antitumor activity of drugs alone and in combination was evaluated in human breast cancer xenografts established by subcutaneous injection of 1833/TGL cells. A suspension of 7.5×10^6^ tumor cells in 200 µL of PBS was injected subcutaneously into the left flank of female 6-week old nude mice; when tumors reached ∼70 mm^3^, mice were divided in 4 groups (n = 9–10) and treated with drugs or vehicle. Tivantinib was administered orally at the dose of 300 mg/kg in a volume of 10 mL/kg of body weight daily until the end of the experiment. Control mice were treated every day with vehicle (PEG 400∶20% vitamin E in TPGS solution [60∶40]) in an equal volume. ZA was administered at the dose of 100 mg/Kg every 72 hours by intraperitoneal inoculation.

The diameter of subcutaneously growing tumors was measured with a caliper twice a week until the animals were sacrificed after 27 days of treatment. Tumor weight was calculated by the formula: Tumor weight (mg) = (length×width^2^)/2. Body weights were measured weekly during the treatment period.

### Experimental Bone Metastasis Model

The bone metastasis experiments in mice were conducted as previously described [10] using 1833/TGL cells, a subpopulation of the human MDA-MB-231 breast cancer cell line that was selected for its high efficiency in metastasizing to bone after intracardiac inoculation [32]. On day 0, 1833/TGL cells (5×10^5^ cells in 100 µL of phosphate-buffered saline [PBS]) were injected into the left cardiac ventricle of mice anesthetized with 5% isoflurane. In this model, mice usually develop bone metastases 11–13 days after tumor cell injection [10].

**Figure 1:**
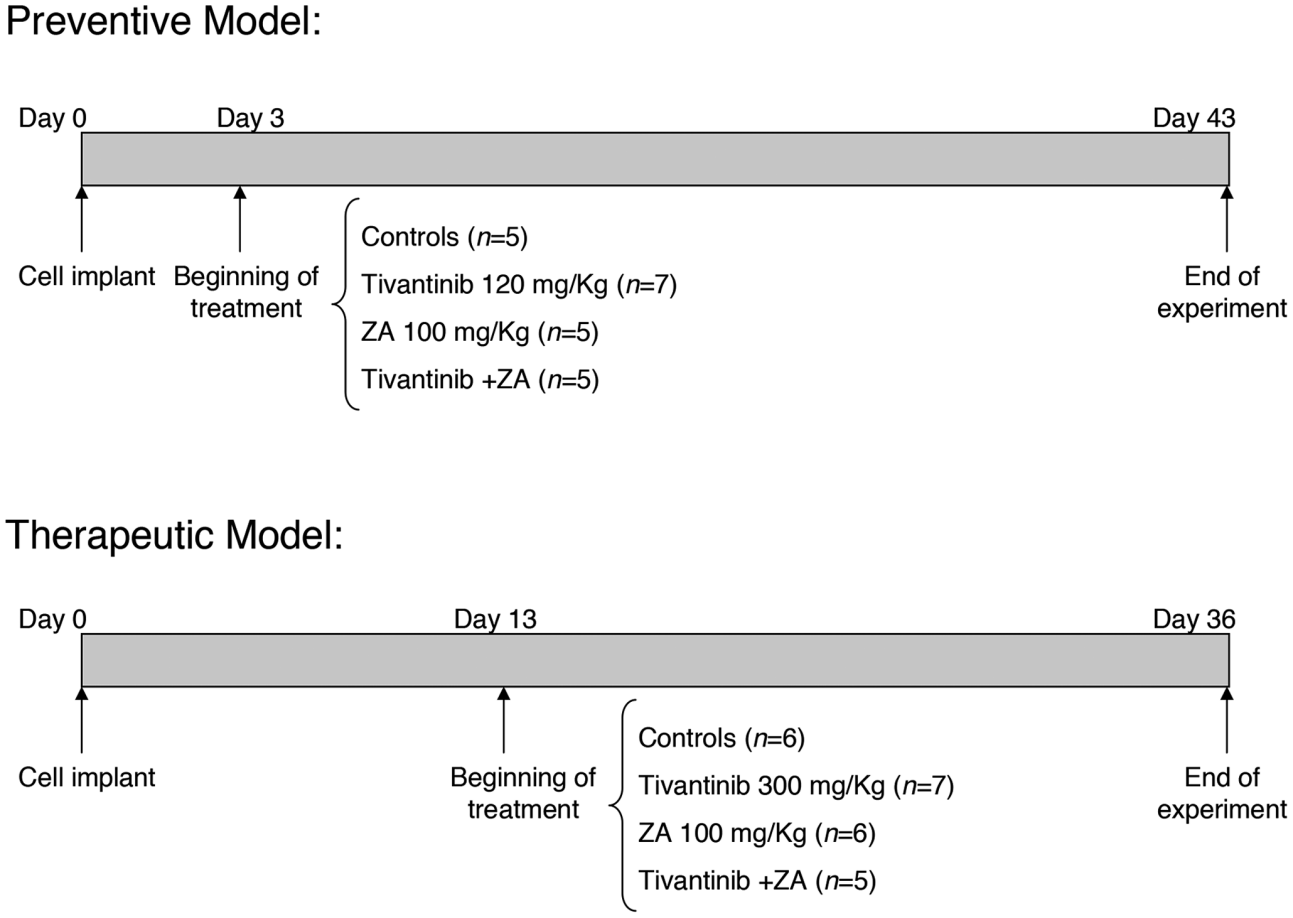
Scheme showing the experimental protocol used.

Two different protocols, “preventive” and “therapeutic”, were used as detailed in Figure 1 and in Methods S1.

### In vivo Bioluminescence Imaging

Noninvasive *in vivo* whole body bioluminescence imaging was performed using the Explore Optix System (ART Advanced Research Technologies). Details are reported in Methods S1.

### Micro-computed Tomography (Micro-CT)

Micro-CT imaging was performed for 3D visualization of osteolytic lesions using an Explore Locus micro-CT scanner (GE Healthcare, London, Canada), without application of contrast agents. Details are reported in Methods S1.

### Bone Histology

Bone histology of bone tissue sections was performed as previously described [10]. Briefly, vehicle and bisphosphonate or/and tivantinib-treated tumor-bearing mice were sacrificed on days 24 and 27 after tumor cell inoculation for preventive and therapeutic protocols, respectively, and both hindlimbs from each animal were dissected, fixed in 4% formalin, dehydrated in 70% (vol/vol) ethanol for 15 minutes, decalcified in Mielodec (Bio-Optica, Italy) for 4 days and at last embedded in paraffin. A microtome (LeicaRM 2255) was used to cut 5 µm-thick sections of decalcified long bones, and the sections were stained with H&E for routine histological examinations.

### Statistical Analysis

All data were analyzed with the use of GraphPad Prism software (version 5.03). In particular, the differences between the two experimental groups in the pattern of the curves relating to body weight and signal intensity of bioluminescence, were evaluated using Student’s t-test, otherwise differences between multiple groups through an ANOVA test one-way followed by the Bonferroni’s test. The analysis of survival of the animals were performed by Log-rank test. *In vitro* data were expressed as the mean ± standard deviation (SD), *in vivo* data as the mean ± standard error (SE). The differences were considered statistically significant at a level of p<0.05.

## Results

### Effects of Tivantinib and ZA on the Formation of Breast Cancer Bone Metastases (“Preventive Protocol”)

**Figure 2:**
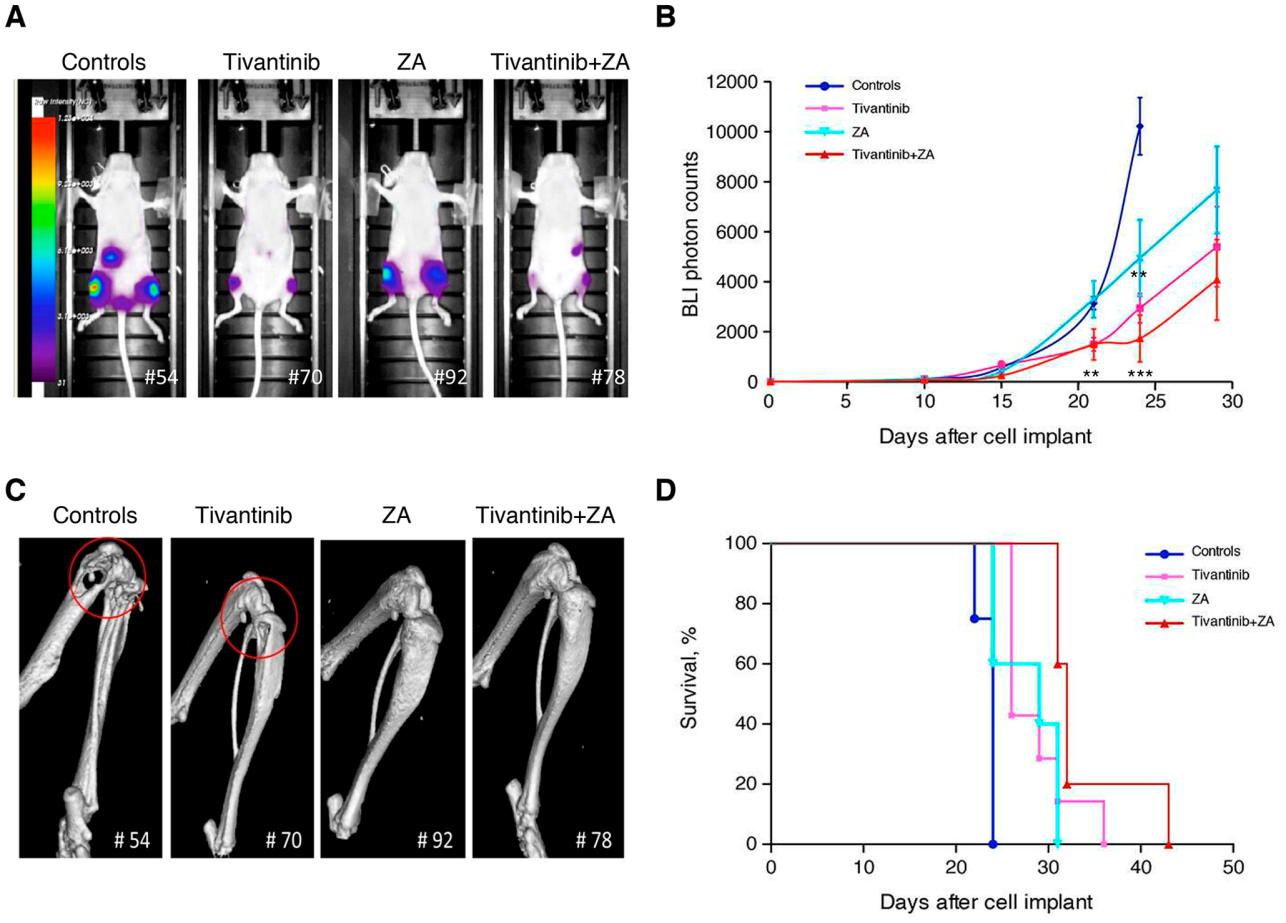
*In vivo* effects of tivantinib and ZA single agent and in combination in a preventive schedule of bone metastases. (**A–B**) *In vivo* bioluminescence imaging (BLI) of 1833/TGL-i.c. injected athymic nude mice (4 week old) treated with tivantinib (120 mg/Kg; *n* = 7), ZA (100 mg/Kg; *n* = 5) and tivantinib+ZA (*n* = 5). (**A**) Ventral images from a representative nude mouse for each group 24 days after implant. Pseudocolor scale bars are consistent for all images of ventral views, in order to show relative changes at metastatic sites over time. (**B**) Average growth of bone metastasis in the hindlimbs of controls and treated mice: photon counts from the hindlimb (right and left) regions of tumor-bearing mice were quantified and displayed over time. The data are presented as mean ± SE. **, p<0.005; ***, p<0.0001. (**C**) The presence of tumor-induced osteolytic lesions were detected by weekly micro-CT scans. Representative 3D reconstruction of micro-CT images of the hindlimbs of controls and treated mice at day 24 after xenografting are reported. Similar results were obtained in the second experiment conducted independently. Red circles indicate osteolysis, the circle’s size was proportional to the extent of bone lesion. Numbers at the bottom of the images represent the number of mice. (**D**) Kaplan-Meier survival plot of the survival rate of 1833/TGL xenografted controls and treated-mice.

We compared the effects of the daily doses of tivantinib as single agent or in combination with ZA on the formation of breast cancer bone metastases by using a preventive protocol in which the drugs were administered at day 3 after tumor cell inoculation and before the establishment of bone metastases (Figure 1). As reported in Figure 2A, showing representative BLI images taken at day 24 after implant, the animals treated with tivantinib exhibited a significant inhibition of tumor growth, as indicated by a reduction in luciferase-related signal in the leg bones when compared to the control group. Tivantinib plus ZA administration markedly reduced the tumor-related BLI signal intensity in the area of the hindlimbs compared to the other experimental groups. Mean value of BLI photon counts relative to metastatic tumor growth in the hindlimbs of controls and treated mice are shown in Figure 2B. An exponential increase of photon emission associated with an increase in bone tumor burden was clearly evident from day 14 onward in the hindlimbs of control mice. As compared to photon counts detected in control mice, preventive treatment with tivantinib alone or in combination with ZA resulted in a significant reduction of bioluminescence signal starting 21 days after implant (p = 0,0044, control vs tivantinib; p = 0,042, control vs tivantinib+ZA). At day 24 after implantation, this inhibitory effect was maintained, the combination being much more effective than tivantinib in delaying metastatic tumor growth in the bone (p = 0,0036, control vs Tivantib; p = 0,0007, control vs tivantinib+ZA) (Figure 2B). ZA decreased mammary tumor burden in the bone 24 days after tumor cell implant, but this reduction was not statistically significant.

The effect of tivantinib alone and in combination with ZA on 1833/TGL-induced osteolysis was also assessed by micro-CT analysis (Figure 2C). Three-dimensional reconstructed micro-CT images of the hindlimbs of representative control animals revealed severe bone destruction on day 24 following cancer cell transplantation. In contrast, bones from 120 mg/kg tivantinib-receiving mice showed only limited signs of osteolysis. ZA administration alone completely inhibited radiologically evident osteolytic lesions, hiding any tivantinib-related effect.

**Figure 3:**
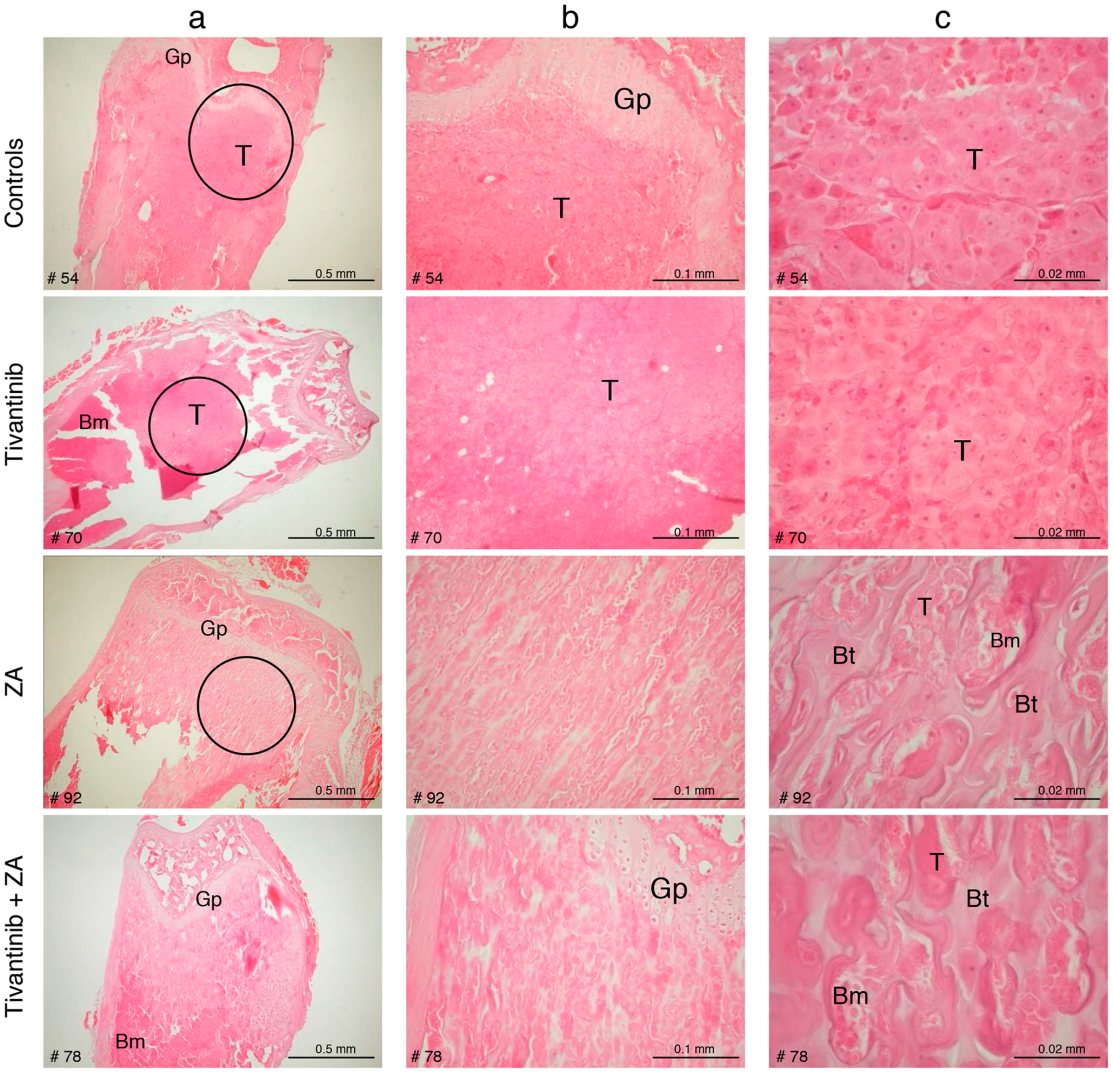
Ex vivo histological analysis. Representative H&E-stained sections of femur and tibia from vehicle and tivantinib or/and ZA treated mice at day 24 from implant are shown. For each group, five sections from five different mice were analyzed. Particular regions of bone from controls and treated mice bordered by a circle in the panels of columns “a” have been magnified in the middle “b” panels and right “c” panels (20X and 100X magnification, respectively). T, metastatic tumor mass; Bm, bone marrow; Bt, bone trabeculae; Gp, growth plate. Numbers at the left bottom of the images represent the number of mice.

The effects of the combination on morbidity, assessed by changes in body weight, and mortality were evaluated. As shown in Figure S1, mice in the tivantinib and tivantinib+ZA groups increased their body weight till day 21, whereas the mice in the control and ZA groups began losing weight at day 15. Consistent with this observation, tivantinib, alone or in combination with ZA, significantly improved the survival rate of mice (Figure 2D). The median survival of controls and tivantinib+ ZA mice was 24 and 32 days, respectively. All mice in the control group died within 24 days from implant, whereas in the tivantinib+ZA group one animal was still alive at day 43. There was not a statistically significant difference between the survival of the control and ZA alone groups. Histological examination performed at the end of the experiment corroborated BLI and micro-CT findings (Figure 3). In particular, H&E staining of tibial and femoral sections from control mice confirmed the massive presence of metastatic 1833/TGL-derived tumor masses within the bone marrow cavity. In tivantinib-treated mice, there were fewer and smaller bone metastases than in control mice. In both ZA and tivantinib+ZA groups, a significant increase of trabecular bone density, as consequence of zoledronic acid treatment, was observed. This result was in line with the micro-CT images showing that in both these groups the integrity of bone was preserved and osteolysis was prevented. The bone metastatic tumor cells growing in the presence of ZA were embedded in the trabecular bone (Figure 3).

### Effects of Tivantinib and ZA on the Progression of Breast Cancer Bone Metastases (“Therapeutic Protocol”)

**Figure 4:**
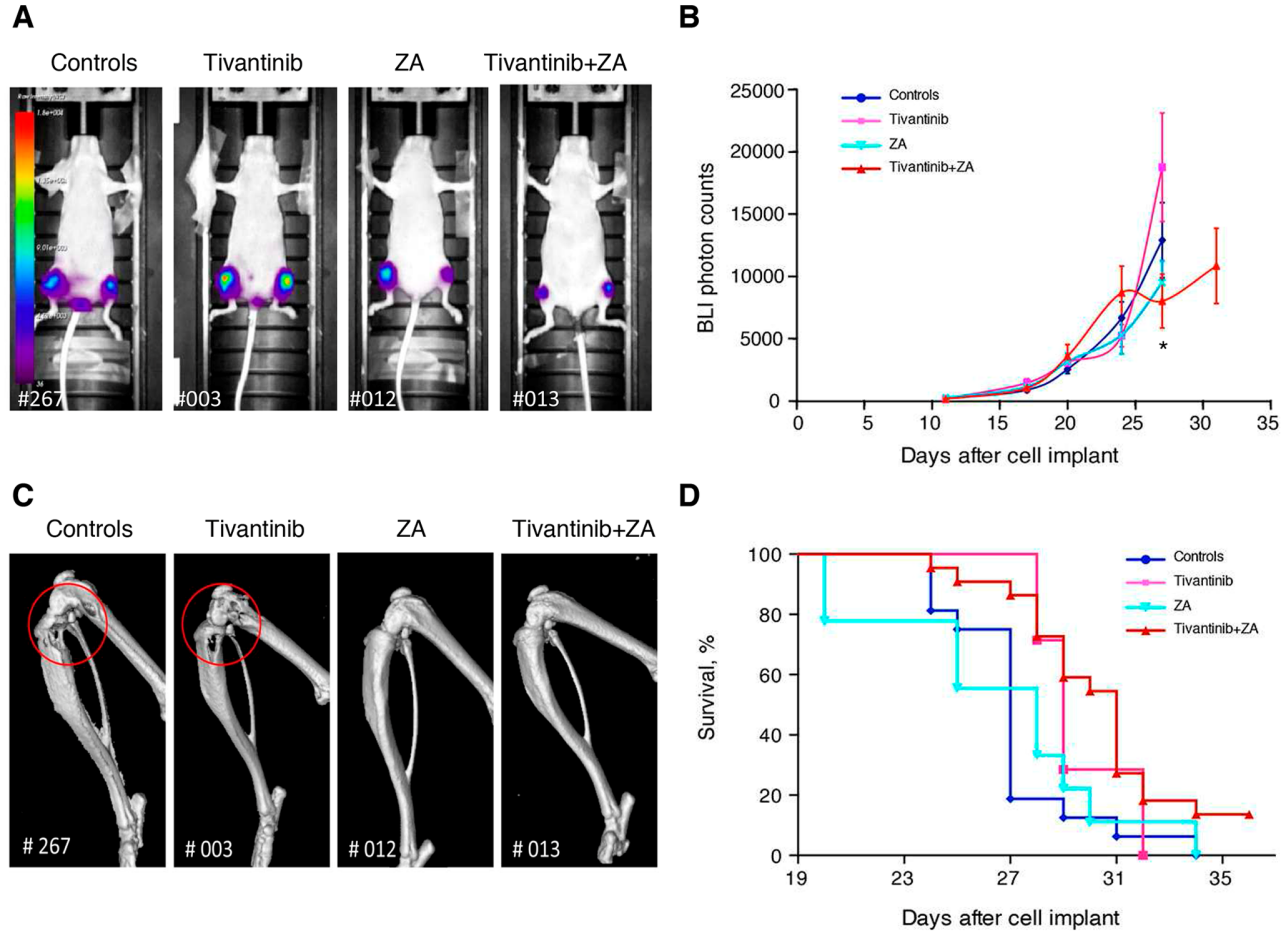
*In vivo* effects of tivantinib and ZA alone and in combination in a therapeutic schedule of bone metastases. The bone metastatic 1833/TGL cells were injected into the left cardiac ventricle of 4-week old athymic nude mice (5×10^5^ cells/mouse). When bone metastases were detectable (11 days after cell implant), implanted animals were randomized into 4 groups of 10 mice each: controls treated with vehicle PEG 400∶20% vitamin E TPGS solution (60∶40) or treated groups administered with tivantinib alone (300 mg/Kg), ZA (100 mg/Kg) alone and combination of tivantinib plus ZA. Both vehicle and tivantinib were administered daily *per os* starting 13 days after implant until the end of the experiment. ZA was administered i.p. every 2 days starting 13 days after implant till the end of the experiment. (**A**) Representative optical scanning in the supine position for each group 27 days after tumor implant. The intensity of the BLI signal, measured as total photon flux, is shown as a pseudo-color scale bar. Numbers at the left bottom of the images represent the number of mice. (**B**) At the indicated times after xenografting, the BLI signal was captured and the growth kinetics of right and left hindlimb metastasis for each group were expressed in the graph as mean value of total photon counts from the hindlimbs of animals. Data are presented as mean value ± SE; *, p<0.05 tivantinib+ZA vs tivantinib. (**C**) Representative volume-rendered micro-CT images of osteolytic bone metastases obtained 27 days after xenografting are shown. Red circles indicate osteolysis and the numbers at the bottom of the images represent the number of mice. (D) Kaplan–Meyer curves of vehicle and treated groups.

The therapeutic protocol was applied to determine if tivantinib inhibits the growth of well established bone metastases. Drugs were administered to 1833/TGL-injected mice 13 days after cell implant, when bone metastases were already detectable by BLI technique. Bone metastases progression was measured by weekly bioluminescence optical imaging analysis. BLI images of representative animals from different experimental groups are shown in Figure 4A and the overall results of treatment are reported in Figure 4B. Bioluminescent emissions were detected in all injected-mice starting 11 days after tumor inoculation. From day 11 onward, BLI photon signals, mainly localized in the hindlimbs, started to exponentially increase in all experimental groups. Differently from what had been observed in the “preventive” model, both single agent tivantinib and ZA, given after bone metastases had developed, could not decrease the tumor growth in the hindlimbs of animals. Although at early time points there were no differences among experimental groups, from day 27 onward, the combination of tivantinib plus ZA appeared to be effective in delaying progression of metastatic tumor growth (p value = 0.0464 tivantinib+ ZA vs tivantinib).

Consistent with the optical imaging analysis, we found that, at day 27, tivantinib-treated mice developed osteolytic lesions similar to controls. On the contrary, treatment with ZA, alone or in combination with tivantinib, resulted in the notable attenuation of the tumor-associated osteolysis (Figure 4C). Despite the only limited effect on bone metastatic tumor growth as assessed by BLI, treatment with tivantinib and ZA significantly improved survival (P value = 0.0015, Log-rank test) compared to controls (Figure 4D). Eighty percent of mice in the control group died by day 27 after implant, while all the animals in the combination treatment group were still alive. The median survival time of tivantinib plus ZA-treated mice was 31 days, while control animals had a median survival time of 27 days. Considering that the treatments were started when bone metastasis were well established, the 15% increase in survival represents a significant result, taking into account that 3 out 22 animals of the combination group survived up to 36 days, when the experiment was stopped. By contrast, treatments of tivantinib or ZA alone did not improve the survival rate of bone metastases-bearing mice (median survival was 29 and 28 days, respectively vs 27 days for controls,). Changes in body weight of animals confirmed the efficacy of combination over single agent treatments. In fact, as shown in Figure S2, the relative body weight curve of controls, tivantinib and ZA-treated mice started to decline at day 17–18, consistent with the exponential increase of bioluminescence intensity indicative of the rapid progression of bone metastases. By contrast, body weight of combination-treated animals remained almost unchanged till day 31.

At the end of the experiment, histologic examination of tibiae and femura corroborated the micro-CT findings (Figure S3). In vehicle and tivantinib-treated animals trabecular bone was effaced and replaced by tumor. In contrast, treatment with ZA, alone or in combination with tivantinib, preserved the integrity of bone trabeculae and prevented bone destruction.

### Antitumor Activity of Tivantinib and ZA Combination in a Subcutaneous Mouse Model

**Figure 5:**
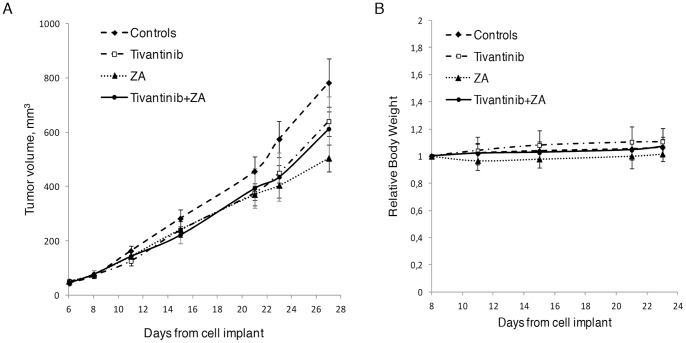
Antitumor activity of tivantinib and ZA alone and in combination against subcutaneous breast cancer xenografts. A breast cancer xenograft model was established using 1833 cells (7.5×10^6^) implanted s.c. into the flanks of female athymic nude mice (6-week old). When tumor volume reached 70 mm^3^ animals were randomized into 4 groups: tivantinib (300 mg/Kg), ZA (100 mg/Kg), tivantinib+ZA-treated mice and controls treated with vehicle (PEG 400∶20% vit. E TPGS solution [60∶40]). Tivantinib and vehicle were administered p.o. in a volume of 10 mL/kg of body weight daily until the end of the experiment. ZA was administered i.p. every 2 days starting 13 days after implant till the end of the experiment. (**A**) Tumor growth inhibition. Tumor size was measured using Vernier caliper twice a week until the animals were sacrificed after 27 days of treatment. Tumor weight was calculated by the formula: Tumor weight (mg) = (length×width2)/2. (**B**) Relative body weight of mice bearing subcutaneous tumor xenografts and treated with vehicle or drugs. Body weights were measured twice weekly. Results are expressed as mean ± SD; *n* = 9–10.

To determine whether the combination of tivantinib and ZA has antitumor activity beyond the setting of bone metastasis, a subcutaneous xenograft model was generated by subcutaneous injection of 1833/TGL cells into the left flanks of athymic nude mice. Chronic treatment with vehicle, tivantinib at 300 mg/kg dose as single agent or in combination with ZA was initiated when tumors were established and reached approximately 70 mm^3^ tivantinib alone had a slight effect in reducing tumor growth and the magnitude of this effect did not change with the addition of ZA (Figure 5A). Daily drug administration was well tolerated by the animals with no significant differences in body weights of animals during the experiment (Figure 5B).

### In vitro Cytotoxic Effects of Tivantinib and ZA

**Figure 6:**
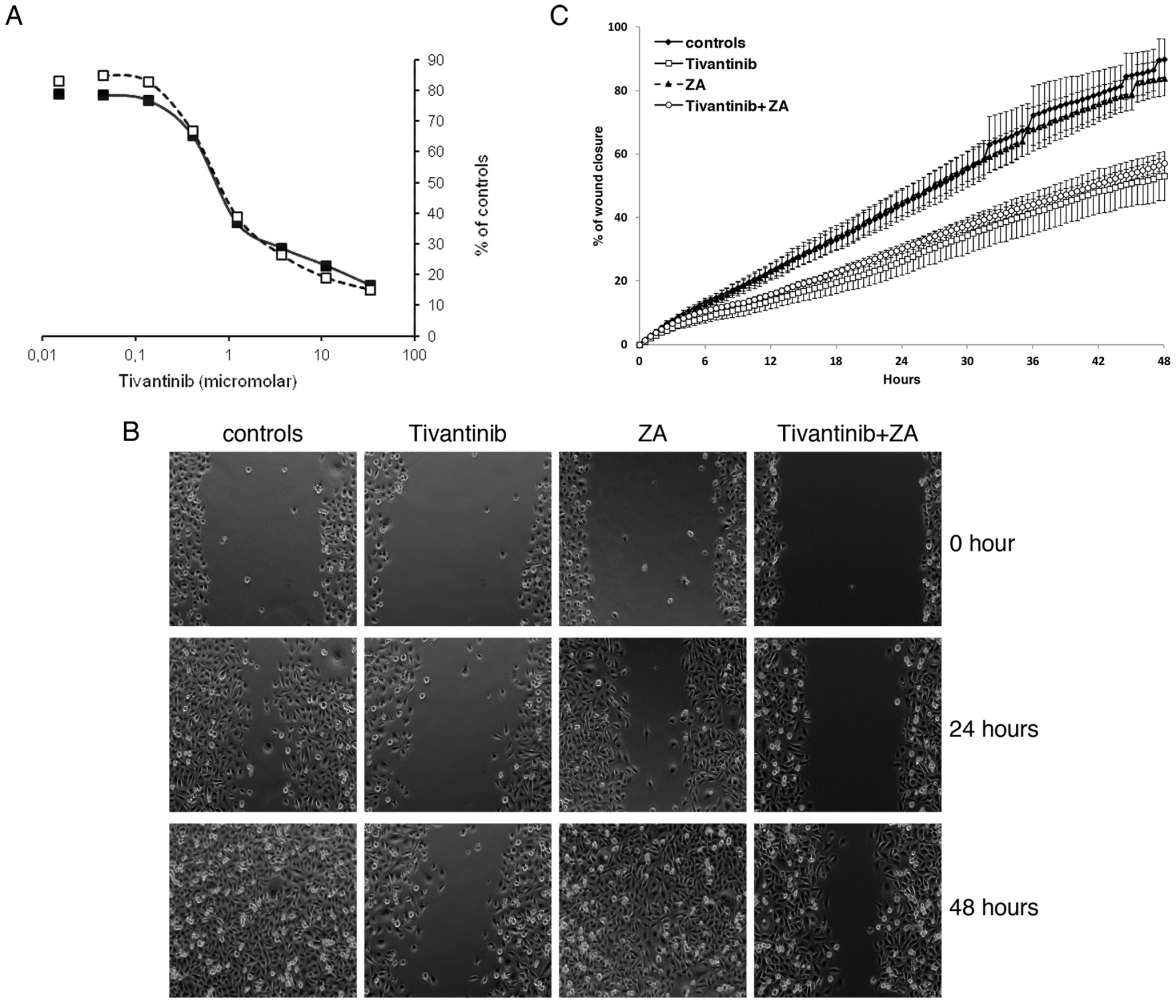
Effect of combination on *in vitro* cytotoxicity and cell migration. (**A**) In vitro cytotoxic activity of tivantinib alone (▪) or in combination with ZA (□) against 1833/TGL cells. Values represent the percentage of controls. (**B**) 1833/TGL cells were seeded at the concentration of 1×10^5^ cells/mL on a 12-well culture plate in six-well plates. On day 0, for each well, a wound was made in the center of the monolayer of confluent cells with a sterile plastic pipette tip and vehicle or drugs were added. The plate was placed under a motorized inverted microscope and wounds were photographed at 0 (*t*_0_), 24, and 48 hours after wounding. Representative photographs of a filed of view from the different experimental conditions are shown. (**C**) Quantitative analysis: the level of cell migration was quantified as the percentage of wound closure at each time point after the wound scratch. Values represent averages ± SE of two independent experiments, each consisting of 2 replicates. Statistical analysis were performed at 24 and 48 hours after wounding.

We assessed the cytotoxic activity of Tivantinib and ZA, alone and in combination, on the growth of 1833/TGL human breast cancer cell lines were tested in vitro. Treatment of cells with tivantinib resulted in a concentration-dependent decrease in cell proliferation which was not significantly increased by the addition of tivantinib (Figure 6A).

### Effect of Tivantinib and ZA on in vitro Tumor Cell Migration

To determine whether the combination of tivantinib and ZA impacts cellular motility the wound-healing migration assay was used. As shown in Figure 6B, reporting photographs of cells migrating into scratch wounds tivantinib alone decreased the migration of 1833/TGL cells compared to the untreated control cells. As confirmed by the quantitative analysis reported in Figure 6C,treatment with tivantinib led to a significant decrease in motility of 1833/TGL cells, which was not modified by the addition of ZA. ZA alone failed to alter the migration pattern of 1833/TGL cells compared to control conditions.

## Discussion

The formation of bone metastases is a multistep event regulated not only by cancer cells but also by the host microenvironment. Interaction between tumor cells and bone microenvironment creates a vicious cycle that disrupts normal bone homeostasis and leads to tumor growth in bone.

Molecules like biphosphonates (particularly ZA) or RANKL antibodies (denosumab), through their ability to interfere with osteoclastogenesis, represent the current standard therapeutic option for patients with bone metastasis [25], [33], [34], [35], [36]. Phase III trials comparing denosumab versus ZA seem to indicate a superiority of denosumab in reducing the scheletal related events (SREs) in patients with metastatic cancers (including breast cancer patients) [37], [38], [39]. Whatever the treatment and its role in the adjuvant setting, it is clear that additional therapeutic strategies are needed.

We have previously shown the involvement of the receptor tyrosine kinase MET and its ligand HGF (also known as scatter factor, HGF/SF) in tumor-bone interaction contributing to human breast cancer bone metastases [9]. MET receptor has been shown to be overexpressed and/or mutated in a variety of malignancies, contributing to the invasive growth of cancer. Several MET targeted inhibitors are currently assessed in the treatment of different solid tumors [7], [40]. At present, however, there are no strong clinical data on the efficacy of MET inhibition in treating bone metastases from breast cancer. In a previous work, our group has demonstrated the effectiveness of MET inhibition, by both tivantinib (ARQ 197) and a specific short hairpin RNA (shRNA) against MET, in delaying the onset of experimental breast cancer-derived bone metastases.

In the present study, the effects on bone metastases of tivantinib and ZA in combination were studied. The in vivo antimetastatic effect of tivantinib was increased with the addition of ZA. The combination was superior to both single agents in terms of control of the growth in the bones and survival. The combination was effective against bone metastasis but not on the primary tumor growing subcutaneously or when the same cells were treated in vitro.

Although ZA has been reported to have a direct cytotoxic activity in vitro against cancer cells and in patients in neoadjuvant setting in combination with chemotherapy [41], [42], [43], its main mechanism of action is related to targeting osteoclasts as shown by the protective effect against osteolysis. Tivantinib and ZA have therefore a different mechanism of action and they likely hit different target cell populations, i.e cancer cells and osteoclasts, respectively. This represents an ideal situation for a combination treatment approach. Indeed our results showing a superiority of the combination of tivantinib and ZA versus single agents in the inhibition of bone metastasis, make unlikely a potential direct action of ZA on cancer cells.

This of course does not rule out the possibility that ZA can directly inhibit cancer cell growth but rather indicate that additivity of the combination of tivantinib and ZA can be achieved in conditions in which ZA does not exert direct antitumor activity, again supporting the idea that a co-targeting of cancer and host cells represents a valuable option.

In the adjuvant setting the use of ZA has proven efficacious in delaying SREs associated with breast cancer [44], [45]. In general, bone targeting therapies have only limited palliative effects in the treatment of advanced, metastatic breast cancer. New combination therapies in the treatment of, i.e. (bone) metastatic breast cancer are needed. The results of our study indicate that, at least in this experimental setting, the combination of a c-met inhibitor (tivantinib) and ZA potentiates the antimetastatic activity of tivantinib without increasing the toxicity. The increase in survivals of animals with bone metastasis achieved with the combination both when the treatment started at early stages and, to a lower extent, when bone metastasis were already measurable, suggests that the two drugs could have additive activity both in the prevention of bone metastases and in the control of bone tumor growth.

## Supporting Information

Figure S1 **In vivo effect of preventive treatments on body weight of bone metastases-bearing mice.** After intracardiac injection of 1833/TGL cells, animals’ body weights were measured weekly till the end of the experiment. RBW was calculated as RBW = Bt/B0, where Bt is body weight at the day of measurement and B0 is body weight at the day of tumor cell injection. Data are presented as mean value of relative body weight ± SE as function of days after cell implantation.
(TIF)

Figure S2 **In vivo effect of drug combination on body weight of 1833-injected mice in a therapeutic setting.** Subconfluent 1833/TGL cells were trypsinized, washed, resuspended in PBS to a final concentration of 5×105 cells/100 µL, and finally injected into the left cardiac ventricle of anesthetized athymic nude mice (4-week old). When bone metastases were established, as assessed by BLI, injected animals were randomized into 4 groups: vehicle, tivantinib 300 mg/kg alone, ZA (100 mg/Kg) or tivantinib plus ZA. Body weights were measured weekly during the treatment period. Relative body weight (RBW) of control and treated mice was calculated as RBW = Bt/B0, where Bt is body weight at the day of measurement and B0 is body weight at the day of tumor cell injection. Data are presented as mean value of relative body weight ± SE and plotted in graph as function of time after cell implantation.
(TIF)

Figure S3 **Ex vivo histological analysis.** Representative H&E-stained sections of femur and tibia from vehicle and therapeutic protocol of tivantinib or/and ZA treated mice at day 24 from implant are shown. For each group, four sections from three different mice were analyzed. Particular regions of bone from controls and treated mice bordered by a circle in the panels of columns “a” have been magnified in the middle “b” panels and right “c” panels. T, metastatic tumor mass; Gp, growth plate; Bt, bone trabeculae. Numbers at the left bottom of the images represent the number of mice.
(TIF)

Methods S1 **Supplementary methods file containing the following information.** In vitro wound-healing assay; Experimental Bone Metastasis Model; In vivo Bioluminescence imaging; Micro-computed tomography (micro-CT).
(DOC)
