# Supplementary figures and images for: Combination of the c-Met Inhibitor Tivantinib and Zoledronic Acid Prevents Tumor Bone Engraftment and Inhibits Progression of Established Bone Metastases in a Breast Xenograft Model
Source: PLoS One. 2013 Nov 18;8(11):e79101. doi: 10.1371/journal.pone.0079101 (PMC3832513; doi:10.1371/journal.pone.0079101)

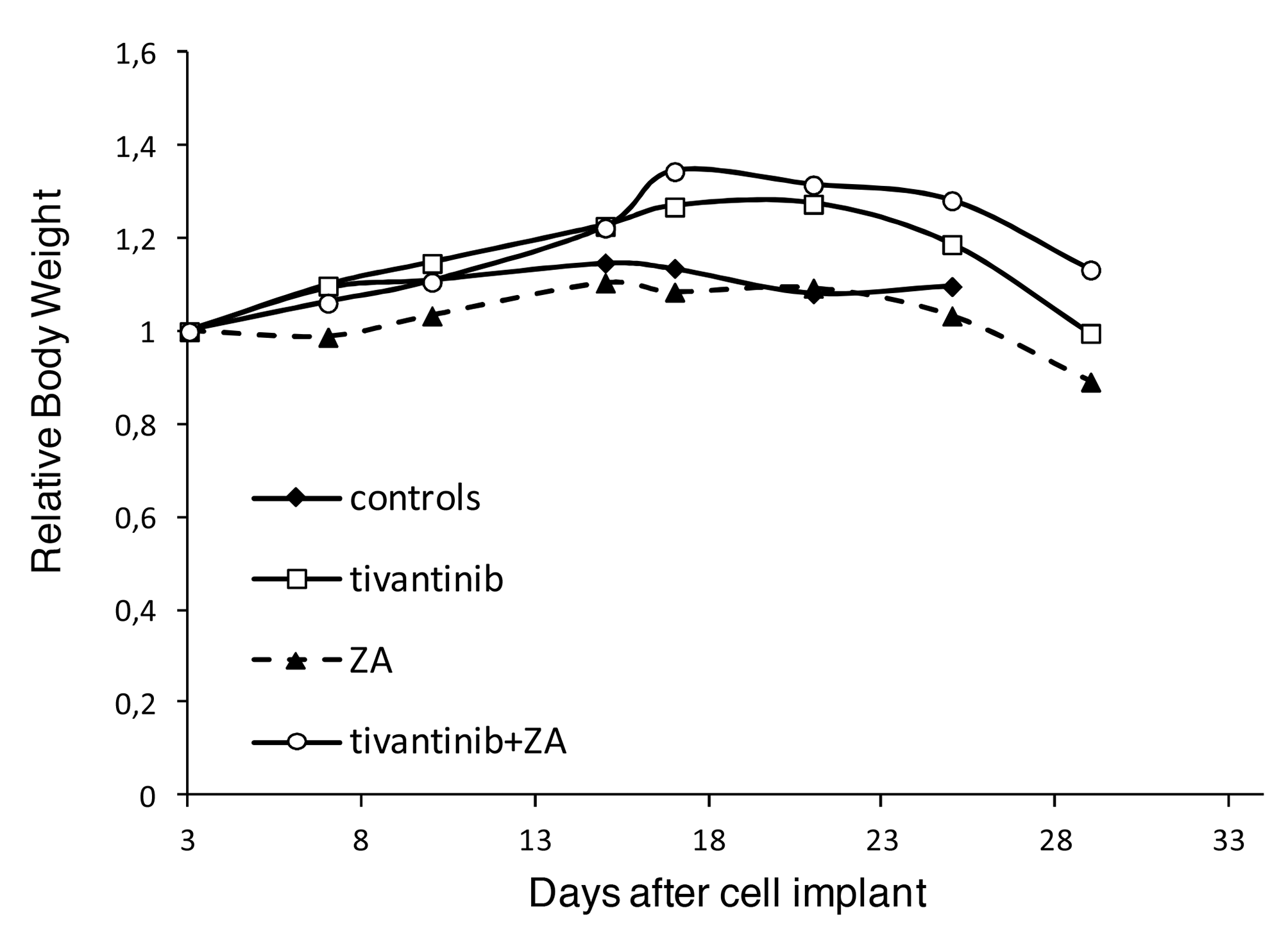

Supplement: Figure S1 — In vivo effect of preventive treatments on body weight of bone metastases-bearing mice. After intracardiac injection of 1833/TGL cells, animals’ body weights were measured weekly till the end of the experiment. RBW was calculated as RBW = Bt/B0, where Bt is body weight at the day of measurement and B0 is body weight at the day of tumor cell injection. Data are presented as mean value of relative body weight ± SE as function of days after cell implantation. (TIF) [file pone.0079101.s001.tif]

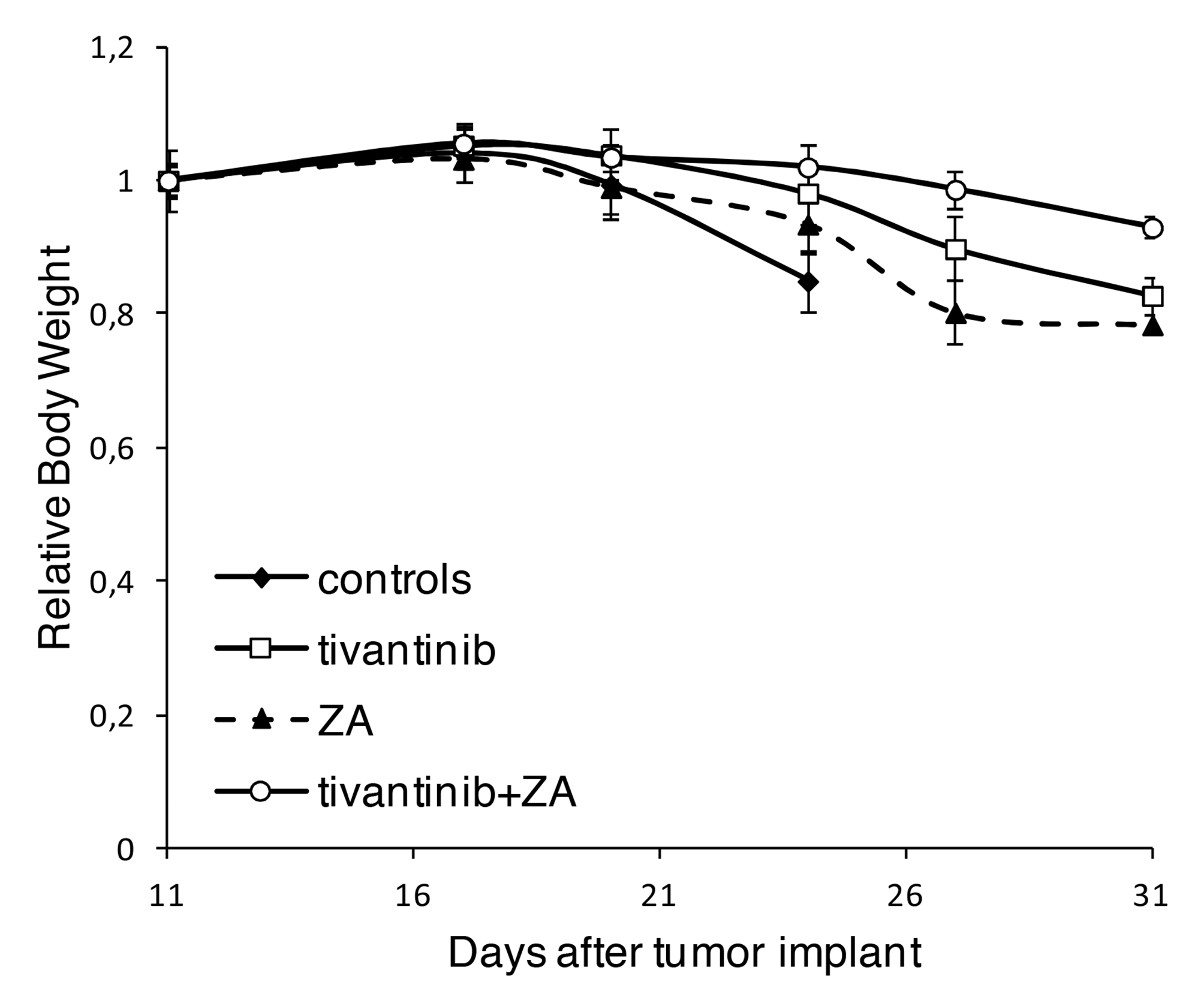

Supplement: Figure S2 — In vivo effect of drug combination on body weight of 1833-injected mice in a therapeutic setting. Subconfluent 1833/TGL cells were trypsinized, washed, resuspended in PBS to a final concentration of 5×10^5^ cells/100 µL, and finally injected into the left cardiac ventricle of anesthetized athymic nude mice (4-week old). When bone metastases were established, as assessed by BLI, injected animals were randomized into 4 groups: vehicle, tivantinib 300 mg/kg alone, ZA (100 mg/Kg) or tivantinib plus ZA. Body weights were measured weekly during the treatment period. Relative body weight (RBW) of control and treated mice was calculated as RBW = Bt/B0, where Bt is body weight at the day of measurement and B0 is body weight at the day of tumor cell injection. Data are presented as mean value of relative body weight ± SE and plotted in graph as function of time after cell implantation. (TIF) [file pone.0079101.s002.tif]

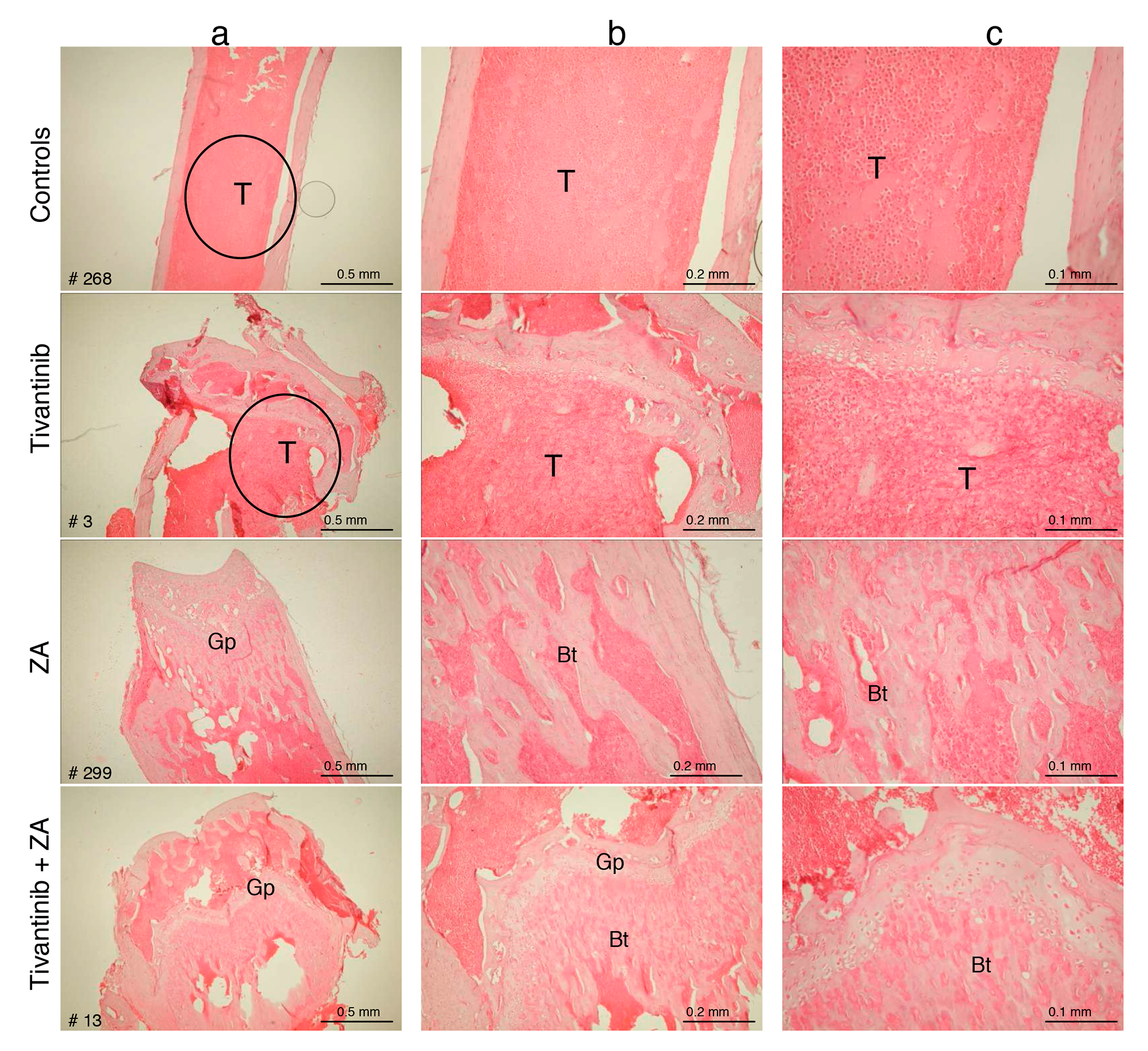

Supplement: Figure S3 — Ex vivo histological analysis. Representative H&E-stained sections of femur and tibia from vehicle and therapeutic protocol of tivantinib or/and ZA treated mice at day 24 from implant are shown. For each group, four sections from three different mice were analyzed. Particular regions of bone from controls and treated mice bordered by a circle in the panels of columns “a” have been magnified in the middle “b” panels and right “c” panels. T, metastatic tumor mass; Gp, growth plate; Bt, bone trabeculae. Numbers at the left bottom of the images represent the number of mice. (TIF) [file pone.0079101.s003.tif]
