# Supplementary material for: Combination of the c-Met Inhibitor Tivantinib and Zoledronic Acid Prevents Tumor Bone Engraftment and Inhibits Progression of Established Bone Metastases in a Breast Xenograft Model
Source: PLoS One. 2013 Nov 18;8(11):e79101. doi: 10.1371/journal.pone.0079101 (PMC3832513; doi:10.1371/journal.pone.0079101)
Supplement: Methods S1 — Supplementary methods file containing the following information. In vitro wound-healing assay; Experimental Bone Metastasis Model; In vivo Bioluminescence imaging; Micro-computed tomography (micro-CT). (DOC) [file pone.0079101.s004.doc]

**SUPPLEMENTARY METHODS**

**In vitro wound-healing assay**

To visualize and photograph migration of cells into the wounded area, the plate was placed under an IX81 motorized inverted microscope (Olympus) fitted with an incubator to maintain 37°C, 5% CO_2_, and 60% humidity (OKOlab). Cells were followed for 48 hours, and every 30 minutes an image was captured using an ORCA-ER CCD camera (Hamamatsu) connected to the microscope. Phase-contrast pictures were analyzed by ImageJ software (Wayne Rasband, USA). The area of denuded surface was quantified immediately after wounding and for the entire duration of the experiment. The extent of wound closure was determined by calculating the ratio between the surface area of the wound for each time point and the surface of the initial wound.

**Experimental Bone Metastasis Model**

Two protocols were used:

A "preventive" protocol in which the drugs were administered starting from day 3 after intracardiac transplantation. Vehicle (PEG 400: 20% vitamin E TPGS solution (60:40)) and tivantinib were administered chronically, by gavage, at a dosage of 120 mg/Kg from day 3 until the end of the experiment. ZA was injected intraperitoneally from day 3 as well at the dose of 100 mg/Kg every 72 hours for the duration of the experiment.

A "therapeutic" protocol where drug treatments started from day 13. Tivantinib was chronically administered at a dosage of 300 mg/kg by gavage and ZA every 72 hours by intraperitoneal inoculation.

For both protocols, the weight of the animals was monitored twice weekly for the duration of the experiment. During the autopsy the bones of the hind legs, especially the tibia and femur, were removed and treated for subsequent histological analysis.

**In vivo Bioluminescence imaging**

Prior to acquiring images, the substrate firefly D-luciferin at a dose of 150 mg/Kg body weight in sterile water was administered by intraperitoneal injection to anesthetized (5% isofluorane) animals and allowed to distribute for 10 min.

Mice were then placed onto the warmed (37°C) stage inside the camera box and 2-3% isofluorane anesthesia was maintained using a nose cone delivery system for the duration of image acquisition. For each animal, at different time points after tumor cell injection, as detailed in the figures, a region of interest (ROI) was designed over the hindlimbs of mouse, and light emission in the selected ROI was quantified as total photon counts using Optiview software (version 2.02.00; ART Advanced Research Technologies). Photon emission was recorded as pseudo-color images representing the spatial distribution of detected photon counts emerging from active luciferase within the animal. Overlay of the gray-scale (body reference photograph) and pseudo-color images allowed localization of metastasis within the animal. The mice were imaged weekly; ventral views facing the camera were acquired because the ventral imaging position was found to be most sensitive to detect bone metastasis in the leg region of implanted mice. For each group and for each time point, the mean value of BLI signal detected around the hindlimbs (both right and left) of mice was calculated and plotted against days after cell inoculation in a histogram or in a linear graph.

**Micro-computed tomography (micro-CT)**

Mice were anesthetized with a continuous flow of 3% isoflurane/oxygen mixture and were scanned once a week during the experiment. A micro-CT lower-resolution (Bin-4) protocol was performed using 80 kV, 450 μA with 100 msec per projection and 400 projections over 360° for a total scan time of approximately 10 minutes. The isotropic resolution of this protocol is 93 μm. The radiation dose from this protocol was estimated to be 0.11 Gy. The reconstructed 3D images were viewed and analyzed using MicroView analysis software (version 2.1.2; GE Healthcare). A volume of interest (VOI) around the hindlimb of mice was designed and bone structure was separated from air and soft-tissues with the help of a global thresholding procedure. Once defined the optimal threshold for bone, isosurface volume rendering function (=a raycast method where surfaces of similar density objects are rendered, and the remaining materials are hidden) was applied to visualize and determine the presence and the extent of tumor-induced osteolytic lesion over time.
